# Susceptibility to Acaricides and the Frequencies of Point Mutations in Etoxazole- and Pyridaben-Resistant Strains and Field Populations of the Two-Spotted Spider Mite, *Tetranychus urticae* (Acari: Tetranychidae)

**DOI:** 10.3390/insects12070660

**Published:** 2021-07-20

**Authors:** Hyun-Na Koo, Jihye Choi, Eungyeong Shin, Wonjin Kang, Sun-Ran Cho, Hyunkyung Kim, Bueyong Park, Gil-Hah Kim

**Affiliations:** 1 Department of Plant Medicine, College of Agriculture, Life and Environment Sciences, Chungbuk National University, Cheongju 28644, Korea; hyunnakoo@hanmail.net (H.-N.K.); cjh5767@gmail.com (J.C.); s2ek04657@gmail.com (E.S.); kwonjin1134@naver.com (W.K.); wonfight@naver.com (S.-R.C.); nshk0917@hanmail.net (H.K.); 2 Crop Protection Division, National Institute of Agricultural Science, Wanju 55365, Korea; florigen1@korea.kr

**Keywords:** *Tetranychus urticae*, etoxazole, pyridaben, point mutation, resistance

## Abstract

**Simple Summary:**

*Tetranychus urticae* Koch is a difficult-to-control pest due to its short life cycle and rapid resistance development. Strains exhibiting resistance to etoxazole or pyridaben exposure have been identified over the past 16 years We collected 8 *T. urticae* field populations from Korea. The resistance ratios of the etoxazole- and pyridaben-resistant (ER and PR) strains were significantly higher than the susceptible strain. The ER and PR strains showed cross-resistance to several acaricides. In addition, the point mutations of the target site were detected in resistant populations.

**Abstract:**

The two-spotted spider mite *Tetranychus urticae* Koch is a major agricultural pest worldwide and is known to rapidly develop resistance to pesticides. In the present study, we explored a field strain that was collected in 2000 and 2003 and has been exhibiting resistance to etoxazole and pyridaben over the last 16 years. The resistance ratios of the etoxazole- and pyridaben-resistant strains (ER and PR) to etoxazole or pyridaben were more than 5,000,000- and 4109.6-fold higher than that of the susceptible strain, respectively. All field-collected populations showed resistance to etoxazole and pyridaben. The ER and PR strains showed cross-resistance to several acaricides. Both I1017F and H92R point mutations were detected in 7 out of 8 field groups. Spirodiclofen and spiromesifen resulted in more than 77.5% mortality in the 8 field groups. In addition, the genotype frequency of the I1017F point mutation was 100.0% in the ER strain, and that of the H92R point mutation was 97.0% in the PR strain. All of the field populations were found to have a high frequency of I1017F. These results suggest that the observation of resistance patterns will help in designing a sustainable IPM program for *T. urticae*.

## 1. Introduction

Tetranychids or spider mites belong to a family of phytophagous mites that cause severe economic effects on agriculture [1]. In Korea, *Tetranychus urticae* has been known as an important pest and has been causing economic damage since the 1950s [2]. *Tetranychus urticae* control is largely based on the use of acaricides in the field; therefore, it rapidly develops resistance due to its short life cycle and high reproductivity [3,4,5,6]. Additionally, resistance of *T. urticae* to various acaricides has become a problem in South America, North America, Asia, Austria, and Europe [7,8,9,10]. Among the acaricides to which this pest has developed resistance, etoxazole was developed in the 1980s by Yashima, a company located in Japan, and was commercialized in 1998 [11,12]. Etoxazole is an oxazoline compound and provides effective control during the egg–nymph stage and is an insect growth regulator that exerts insecticidal activity by inhibiting chitin biosynthesis [13]. The target site is glycosyltransferase chitin synthase 1 (CHS1) [13,14]. Etoxazole resistance was first reported in Japan in 1997 before its commercialization [7,15,16,17,18]. Pyridaben was developed at approximately the same time as etoxazole. It is a pyridazine compound and was discovered in 1984 by Nissan Chemical and was commercialized in 1991 [11]. It inhibits NADH:CoQ, oxidoreductase activity, and provides good efficacy against all developmental stages of various mite species [19,20]. In addition, pyridaben was commonly used in 32 countries until 1994 because it is fast-acting, stable, and biodegrades relatively quickly [21]. However, due to the use of many acaricides, METI (Mitochondrial Complex Electron Transport Inhibitor)-resistant *T. urticae* has been reported since the 1990s [22,23]. *Tetranychus urticae* can be resistant to one acaricide or exhibit multiple or cross resistance to various other acaricides [24,25,26]. The I1017F point mutation is located within the last transmembrane helix and was found to be related to etoxazole resistance [7,14]. The H92R point mutation is located within the PSST homolog in mitochondria complex I and was found to be related to pyridaben resistance [27]. Rapid molecular diagnostics for detecting single resistant and multi-resistant populations primarily identify mutations in the *T. urticae* genes associated with acaricide resistance.

The objective of this study was to evaluate the susceptibility to acaricides in etoxazole- and pyridaben-resistant (ER and PR) strains and in field-collected populations of *T. urticae*. Furthermore, the frequencies of the I1017F and H92R point mutations were investigated to identify potential resistance mechanisms.

## 2. Materials and Methods

### 2.1. Mites

The susceptible (S) strain was first collected in 2005. This strain had never been exposed to acaricides. Resistant strains were treated once a week with the LC_30_~LC_50_ of etoxazole or pyridaben. Information on the eight field populations and resistant strains is shown in Table 1. The mites were reared at 25–27 °C with a 16 L: 8 D photoperiod and 40~60% relative humidity. Kidney beans (*Phaseolus vulgaris* L.) were used as a host.

### 2.2. Acaricides

Commercially formulated abamectin (10% SC), acequinocyl (15% SC), bifenazate (13.5% SC), cyenopyrafen (25% SC), cyflumetofen (20% SC), etoxazole (10% SC), fluxametamide (9% EC), pyflubumide (10% SC), pyridaben (20% WP), spirodiclofen (22% WP), spiromesifen (20% SC), and spirotetramat (22% SC) were purchased from a farm supply store (Cheongju, Korea).

### 2.3. Laboratory Toxicology Assays

#### 2.3.1. Eggs

Kidney bean leaf discs (approximately 35 mm in diameter) were used as substrates for oviposition. Ten mated females (2- to 3-day-old) were transferred on the ventral side of a bean leaf disc placed on cotton soaked in water in a Petri dish (60 mm diameter) and given a 24-h period in which to lay eggs. After 24 h, the adults were removed, and the eggs were counted. The leaf discs with eggs were treated with the selected pesticide (1/8-, 1/4-, 1/2-, 1-, 2-, and 4 times the recommended concentration) suspension using a sprayer and were then dried in the dark for 30 min. The recommended concentration of each pesticide was only used to treat the field-collected populations. Control groups were sprayed with distilled water only. All leaf discs were examined daily for 7 days. The numbers of hatched and unhatched eggs were recorded. The dishes were incubated at 25–27 °C under 40–60% relative humidity and a 16 L:8 D photoperiod. All experiments were replicated three times.

#### 2.3.2. Adult Females

Adult females (2 to 3 days old, avg. 20–150/experiment) were transferred to bean leaf discs (35 mm in diameter) on wet cotton wool using a brush. Solutions diluted to various concentrations (1/8-, 1/4-, 1/2-, 1-, 2-, and 4 times the recommended concentration of pesticide) were sprayed (3 mL each) onto the discs, which were then dried in the dark for 30 min. The recommended concentration of each pesticide was only used to treat the field-collected populations. The dishes were incubated at 25–27 °C under 40–60% relative humidity and a 16 L:8 D photoperiod. Mortality was evaluated at 48 h after treatment. Control groups were sprayed with distilled water only, and the control mortality in all tests never exceeded 5%. All experiments were replicated three times.

### 2.4. General Sequencing and Pyrosequencing of CHS1 and PSST in T. urticae

Genomic DNA was extracted from 200 mites from each *T. urticae* strain using the G-spin^™^ Total DNA Extraction Mini Kit (Intron, Seongnam, Korea) according to the manufacturer’s instructions. Approximately 100 ng of DNA was used as template DNA for PCR. The reactions were performed using a PCR Premix kit (HotStart, Bioneer Co., Daejeon, Korea) and the primers listed in Table 2. The resulting PCR products were purified and directly sequenced using Bioneer Co. The pyrosequencing protocol consisted of 45 PCR cycles performed with the forward primer and biotinylated reverse primer at 0.5 μM, each in 20 μL reaction mixture containing 1× Taq enzyme reaction mix (Enzynomics, Daejeon, Korea). The following cycling conditions were used: one cycle at 95 °C for 15 min; 45 cycles at 94 °C for 30 s, 60 °C for 30 s, and 72 °C for 30 s; and a final step at 72 °C for 10 min. The reactions were performed using a PyroGold reagent kit and a PyroMark ID system (Qiagen, Germantown, MD, USA).

### 2.5. Data Analysis

To estimate the parameters of a concentration–mortality line for each leaf-dip bioassay, replicate data were collected and analyzed using the probit model in the SAS program [28]. Two LC_50_ values were considered different at *p* < 0.01.

## 3. Results

### 3.1. Resistance Ratios (RRs) to Etoxazole and Pyridaben

Bioassays were conducted using etoxazole and pyridaben in etoxazole-resistant (ER) and pyridaben-resistant (PR) strains of *T. urticae*. When the eggs were treated with etoxazole, the ER strain showed an RR that was > 5,000,000-fold higher than that of the S strain (Table 3). The RR of PR strain was 9.5-fold. However, the ER strain had a low RR of 3.6-fold to pyridaben. The PR strain had an RR that was over 4109.6-fold higher than the S strain to pyridaben. As already reported, etoxazole had no effect on the adults. Additionally, pyridaben was not treated with concentrations above 400 ppm (4 times recommended dose) because it showed a repellent characteristic towards adults.

### 3.2. Cross-Resistance to 10 Acaricides in the S, ER, and PR Strains

Cross-resistance to 10 acaricides with different modes of action was determined in the S, ER, and PR strains of *T. urticae* eggs and adults. First, eggs from the ER strain of *T. urticae* showed resistance to abamectin, bifenazate, cyenopyrafen, cyflumetofen, fluxametamide, pyflubumide, and spirotetramat, with RRs of 10.6, >10.7, 20.2, >378.7, 10.5, 682.8, and >1612.9, respectively (Table 4). Eggs from the PR strain of *T. urticae* showed resistance to bifenazate, fluxametamide, pyflubumide, and spirotetramat, with RRs of >10.7, 11.4, 54.1, and >1612.9, respectively. Next, adults from the ER strain of *T. urticae* were resistant to cyenopyrafen, cyflumetofen, pyflubumide, and spirotetramat (Table 5). Adults from the PR strain of *T. urticae* had resistance to cyflumetofen, with an RR of 40.6.

### 3.3. Acaricide Susceptibility of 8 Field-Collected Populations

Of the twelve acaricides, spirodiclofen and spiromesifen had acaricidal activity in all of the field population eggs. Spirodiclofen and spiromesifen resulted in mortality rates of more than 77.5% and 82.5%, respectively (Table 6). Twelve acaricides did not cause mortality of more than 80% in all field adults. OC and CJ had mortality rates greater than 90% with fluxametamide, and CJ had mortality rates greater than 90% with abamectin (Table 7). The LC_50_ value for all field populations was more than 500 ppm when they were treated with etoxazole, and the RR was > 25,500 (Table 8). For pyridaben, seven of the eight field populations, CJ being the exception, had LC_50_ values greater than 4000 ppm, and the RR was > 5480. The LC_50_ value for the CJ population was 103.26 ppm, and the RR was 144.5 (Table 9).

### 3.4. Genotypes of Point Mutations (I1017F and H92R)

Using pyrosequencing, the frequencies of I1017F in *CHS1* and H92R in the *PSST* subunit of mitochondrial electron transport complex I were identified (Table 10). The I1017F point mutation was found in the ER strain but not in the S and PR strains. The H92R point mutation in the *PSST* gene was present in the PR strain but not in the S and ER strains. The sequencing results from the eight field populations showed that the I1017F mutation was present in all of the groups. The H92R point mutation was present in seven field populations. Five of them had a homozygous allele for histidine (H). The OC and YI populations showed heterozygous alleles for H and R (arginine). The genotype frequency of a phenylalanine (F) at the 1017th amino acid position of CHS1 was 100% in the ER strain. In the eight field populations, it was 72~99.0%. However, in the S and PR strains, it was 0.0% and 2.0%, respectively. The genotype frequency of an arginine (R) on the 92th amino acid position of *PSST* was 99.0% in the PR strain. However, in the S and ER strains, it was 11.0% and 29.0%, respectively.

## 4. Discussion

The controlling of two-spotted spider mites mainly depends on chemical control methods, and there are many reports on the development of resistance to acaricides due to their short lifespan and high fecundity. In this study, we determined the cross-resistance of ER and PR strains and the complex resistance of the field-collected populations. We also observed the frequency of point mutations in the etoxazole resistance- (I1017F of CHS1) and pyridaben resistance-related gene (H92R of PSST). As a result, the resistance ratio of the ER and PR strains was less than 10, and there was no cross-resistance. In previous studies, ER strains also showed low cross-resistance to pyridaben [30]. Our results were also similar to that research. However, the PR strain was shown to have cross-resistance to etoxazole by Choi et al. [29]. Since the PR strain used in this study is a more carefully selected strain, this result is considered to be more reliable. In addition, both strains showed a high resistance ratio because they had been exposed to etoxazole or pyridaben for a long period of time (more than 16 years), and the possibility of cross-resistance is low (Table 3). In order to find effective acaricides against etoxazole- and pyridaben-resistant strains, we performed sensitivity evaluation using 10 pesticides. As a result of treatment with ER eggs, the pesticides that showed less than 70% acaricidal effect were abamectin, bifenazate, cyflumetofen, fluxametamide, pyflubumide, and spirotetramat. In PR eggs, the pesticides that showed less than 70% acaricidal effect were abamectin, bifenazate, cyflumetofen, fluxametamide, and spirotetramat (Table 4). Abamectin, bifenazate and pyflubumide were less effective for eggs than adults in a recent study [31,32]. P450s are known to be related to cyflumetofen resistance [33]. Additionally, P450s are known to be related to pyridaben and etoxazole resistance, so the ER and PR strains showed cross resistance to cyflumetofen [33,34]. Fluxametamide has a similar mode of action to diamide. Diamides are more effective in larvae than in the eggs of Lepidopterans, and they seem to have worked similarly in *T. urticae* [35]. Spirodiclofen and spiromesifen showed high acaricidal effects in both resistant strains, but spirotetramat, insecticides belonging to the same chemical class, had a low effect. In a previous study, spirotetramat showed an LC_50_ value 34 times higher than that of spirodiclofen in *Panonychus citri* eggs [36]. Since *T. urticae* and *P. citri* belong to the same family, similar results seem to have been obtained. Therefore, acequinocyl, spirodiclofen, and spiromesifen could be used to the control eggs from the ER and PR strains. Additionally, cyenopyrafen was effective against eggs from the PR strain.

When adults from the ER strain were treated with ten acaricides, the mortality due to cyenopyrafen, cyflumetofen, pyflubumide, spirodiclofen, and spirotetramat was less than 70%. Adults from the PR strain showed resistance to spirotetramat (Table 5). Khalighi et al. [37] reported that etoxazole-resistant strains did not have cross-resistance to cyenopyrafen and cyflumetofen. Cyflumetofen and cyenopyrafen show an increased acaricidal effect when P450 activity is inhibited. P450s and glutathione-*S*-transferases have been related to the acaricidal effect of cyflumetofen [33,38]. Pyflubumide is classified in the new subgroup 25B (carboxanilide) in the IRAC (Insecticide Resistance Action Committee) MoA classification as a novel inhibitor of mitochondrial complex II on the respiratory chain and has a similar mechanism to cyenopyrafen and cyflumetofen, so resistance to this compound may have developed via the same factors [39,40]. Spirodiclofen, spiromesifen, and spirotetramat have sterility effects on adult females and ovicidal effects on eggs but do not have a control effect on adults [41]. Therefore, abamectin, acequinocyl, bifenazate, and fluxametamide will be effective in controlling adults that have developed resistance to etoxazole and pyridaben. In a previous report, Marcic et al. showed that the toxicity of spirotetramat to eggs is most likely caused by its residual effect on hatching larvae [42].

We also compared the acaricidal activities of etoxazole and pyridaben in resistant groups and field populations. As a result, all field populations showed resistance to the two acaricides (Table 6 and Table 7). Etoxazole has been used for over 20 years since its registration in Korea in 1998, and pyridaben has been used for nearly 30 years since its registration in Korea in 1992 [30,43]. Resistance to the two acaricides began to be reported in the late 1990s [11,15,22]. All field populations had some level of resistance because resistance has steadily developed with the long-term use of these chemicals [7,16,17,18]. Based on this result, we treated resistant groups and eight field populations with ten different acaricides on. Since the pesticides used in each region may be different, there may be differences in the degree of resistance development to each pesticide. In all of the field populations, spirodiclofen and spiromesifen had an acaricidal effect of more 77.5% in eggs.

The results of the genotype frequency analysis of the H92R point mutation showed that the OC and YI populations had a lower frequency than the other populations, but the bioassays showed that the resistance ratio was more than 5480-fold (Table 9). In this regard, it has been reported that cyflumetofen and cyenopyrafen-resistant mites have cross resistance to pyridaben [37,43]. Additionally, the mutation frequency of the I1017F gene was different for each region, and the resistance ratio was the same by more than 25,500 times. The reason for this is that although there is a difference in the degree of resistance by region, it seems that the same result appeared because it was not treated at a dose of 500 ppm or more. All field populations had resistance to most of the acaricides used in this experiment. Spirodiclofen and spiromesifen showed excellent effects in the eggs of field populations with complex resistance and may be effective in controlling *T. urticae*.

## 5. Conclusions

To control *T. urticae* effectively, the alternation of acaricides is necessary, and indiscriminate pesticide use should be avoided. Before applying an acaricide, the degree of resistance development should be checked. The development of resistance to etoxazole and pyridaben can be diagnosed using the I1017F and H92R point mutations as resistance diagnostic markers. Almost all field populations have multiple resistance genes; therefore, continuous research on the development of resistance is needed.

## Figures and Tables

**Table 1:** Collection data of mite populations included in this study.

Populations		Date Collected	Region	Host	Collection Locations (South Korea)
Lab-selected	Etoxazole resistant (ER)	Aug 2000	Buyeo	Rose	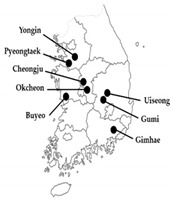
	Pyridaben resistant (PR)	Feb 2003	Uiseong	Rose	
Field-collected	Cheongju (CJ)	Mar 2020	Cheongju	Melon	
	Gimhae-1 (GH-1)	Aug 2019	Gimhae	Rose	
	Gimhae-2 (GH-2)	Aug 2019	Gimhae	Rose	
	Gumi-1 (GM-1)	Aug 2019	Gumi	Rose	
	Gumi-2 (GM-2)	Aug 2019	Gumi	Rose	
	Okcheon (OC)	Feb 2020	Okcheon	Rose	
	Peongtaek (PT)	Mar 2020	Pyeongtaek	Rose	
	Yongin (YI)	Oct 2019	Yongin	Strawberry	

**Table 2:** Primers used for PCR amplification.

Reaction	Target	Name	Sequence
General sequencing	*CHS1*	Demaeght-F	AGATCCTTTACGTCTGGGGC
		Demaeght-R	CAATTGGGACTCGTTTCTTTTCA
	*PSST*	PSST-F	ACAGGTCAGCCAATCGAATC
		PSST-R	ATACCAAGCCTGAGCAGTGG
Pyrosequencing	*CHS1*	CHS-F	GTCTTTTGTAGTGGCGGCATT
		CHS-R	TCCCCAAGTAACAACGTTCAAGT
	*PSST*	PSST-F	TGACTTTTGGATTAGCCTGTTGTG
		PSST-R	AGGACTTGCTCTGAATAACATACCA

**Table 3:** Susceptibility to etoxazole and pyridaben in S, ER and PR strains of *T. urticae*.

Acaricide	Stage	Strain	n	LC_50_ (ppm) (95% CL ^a^)	Slope ± SE	RR ^b^
Etoxazole		S	1780	0.02 (0.02–0.03)	1.95 ± 0.16	1
	Egg	ER	1075	>100,000	-	>5,000,000
		PR	1846	0.19 (0.04–0.67)	2.36 ± 0.45	9.5
		S	266	>100,000	-	No effect
	Adult	ER	107	>100,000	-	-
		PR	93	>100,000	-	-
Pyridaben		S ^c^	462	0.73 (0.31–1.48)	0.91 ± 0.10	1.0
	Egg	ER	1826	2.65 (1.27–4.16)	0.61 ± 0.10	3.6
		PR ^c^	619	>3000	-	>4109.6
		S	90	>400		Not measurable
	Adult	ER	90	>400	-	
		PR	98	>400	-	

^a^ Confidence limits. ^b^ RR, resistance ratio = LC_50_ of resistant strain/LC_50_ of susceptible strain. ^c^ Choi et al. [29].

**Table 4:** Susceptibility to acaricides in the eggs of S, ER, and PR strains of *T. urticae*.

Acaricide	Strain	n	LC_50_ (ppm) (95% CL ^a^)	Slope ± SE	RR ^b^
Abamectin	S	720	0.87 (0.34–2.82)	1.72 ± 0.24	1.0
	ER	496	9.24 (8.71–15.62)	1.64 ± 0.25	10.6
	PR	642	8.52 (5.64–11.84)	1.34 ± 0.38	9.8
Acequinocyl	S	1230	8.02 (6.91–9.53)	2.14 ± 0.19	1.0
	ER	1719	12.59 (4.92–37.54)	1.94 ± 0.51	1.6
	PR ^c^	881	1.47 (0.53–3.10)	1.42 ± 0.21	0.2
Bifenazate	S	1780	65.21 (23.60–136.91)	0.45 ± 0.11	1.0
	ER	2391	>700	-	>10.7
	PR	378	>700	-	>10.7
Cyenopyrafen	S	496	1.68 (0.87–2.63)	2.65 ± 0.47	1.0
	ER	2306	33.85 (20.23–45.74)	1.73 ± 0.26	20.2
	PR	1024	1.32 (0.85–2.54)	1.35 ± 0.28	1.3
Cyflumetofen	S	732	0.79 (0.29–0.99)	1.83 ± 0.31	1.0
	ER	988	>300	-	>379.7
	PR	865	4.57 (1.38–10.32)	1.56 ± 0.19	5.8
Fluxametamide	S	962	4.62 (1.46–14.73)	1.24 ± 0.22	1.0
	ER	477	48.32 (39.12–56.25)	1.56 ± 0.33	10.5
	PR	884	52.64 (27.86–79.19)	2.88 ± 0.37	11.4
Pyflubumide	S	517	0.08 (0.01–0.25)	1.23 ± 0.16	1.0
	ER	727	54.62 (40.07–86.89)	0.94 ± 0.19	682.8
	PR	875	4.33 (2.21–12.36)	1.95 ± 0.14	54.1
Spirodiclofen	S	857	18.23 (12.78–21.38)	1.56 ± 0.28	1.0
	ER	1549	18.02 (12.87–24.33)	2.31 ± 0.13	1.0
	PR	984	19.32 (12.64–32.54)	1.35 ± 1.25	1.1
Spiromesifen	S	909	0.74 (0.38–1.79)	1.85 ± 0.37	1.0
	ER	698	0.33 (0.25–0.43)	1.71 ± 0.16	0.4
	PR	921	0.94 (0.31–2.54)	1.34 ± 0.25	1.3
Spirotetramat	S	873	0.31 (0.08–0.40)	1.22 ± 0.24	1.0
	ER	1733	>500	-	>1612.9
	PR	932	>500	-	>1612.9

^a^ Confidence limits. ^b^ RR, resistance ratio = LC_50_ of resistant strain/LC_50_ of susceptible strain. ^c^ Choi et al. [29].

**Table 5:** Susceptibility to acaricides in adults of the S, ER, and PR strains of *T. urticae*.

Acaricide	Strain	n	LC_50_ (ppm) (95% CL ^a^)	Slope ± SE	RR ^b^
Abamectin	S	134	0.21 (0.08–0.82)	1.27 ± 0.18	1.0
	ER	192	0.10 (0.09–0.12)	1.02 ± 0.15	0.5
	PR	245	0.23 (0.01–0.75)	1.84 ± 0.32	1.1
Acequinocyl	S ^c^	225	2.78 (1.48–6.58)	0.51 ± 0.07	1.0
	ER	545	20.38 (17.31–23.87)	1.72 ± 0.14	7.3
	PR ^c^	225	13.41 (10.06–21.94)	0.92 ± 0.10	4.8
Bifenazate	S	359	5.08 (2.84–10.90)	1.37 ± 0.23	1.0
	ER	363	23.39 (19.44–28.70)	1.19 ± 0.10	4.6
	PR	256	4.76 (2.62–8.29)	1.25 ± 0.33	0.9
Cyenopyrafen	S	182	1.32 (0.54–4.72)	1.37 ± 0.18	1.0
	ER	208	70.61 (54.39–93.28)	1.02 ± 0.13	53.5
	PR	243	6.51 (3.01–17.86)	1.31 ± 0.12	4.9
Cyflumetofen	S	68	0.58 (0.29–0.99)	1.83 ± 0.31	1.0
	ER	212	>1000	-	>1724.1
	PR	135	23.54 (14.81–25.66)	1.35 ± 0.10	40.6
Fluxametamide	S	362	2.26 (1.66–3.09)	2.54 ± 0.32	1.0
	ER	121	1.96 (1.42–5.07)	1.68 ± 0.23	0.9
	PR	253	2.12 (1.73–4.62)	1.26 ± 0.33	0.9
Pyflubumide	S	180	2.75 (1.32–5.31)	1.27 ± 0.14	1.0
	ER	181	>2000	-	>727.3
	PR	183	0.72 (0.34–0.62)	1.16 ± 0.15	0.3
Spirodiclofen	S	67	>1800	-	No effect
	ER	167	>1800	-	-
	PR	108	>1800	-	-
Spiromesifen	S	75	>1000	-	No effect
	ER	131	>1000	-	-
	PR	109	>1000	-	-
Spirotetramat	S	332	32.62 (21.81–57.43)	1.95 ± 0.31	1.0
	ER	221	354.01 (69.44–3345)	0.17 ± 0.06	10.9
	PR	192	262.13 (142.54–1283)	0.67 ± 0.11	8.0

^a^ Confidence limits. ^b^ RR, resistance ratio = LC_50_ of resistant strain/LC_50_ of susceptible strain. ^c^ Choi et al. [29].

**Table 6:** Mortality of *T. urticae* eggs of eight field populations in response to 12 acaricides.

Acaricide	% Mortality ^a^ (mean ± SE)															
	n	CJ	n	GH-1	n	GH-2	n	GM-1	n	GM-2	n	OC	n	PT	n	YI
Abamectin	124	3.4 ± 3.2	135	3.1 ± 3.0	111	3.8 ± 2.5	82	13.0 ± 2.2	82	12.5 ± 5.3	314	11.2 ± 0.1	97	15.1 ± 8.4	219	15.7 ± 1.6
Acequinocyl	86	17.1 ± 3.1	94	9.2 ± 1.5	80	20.0 ± 5.2	101	15.7 ± 3.0	103	11.5 ± 6.0	287	12.3 ± 6.2	79	12.9 ± 5.5	233	18.2 ± 2.2
Bifenazate	108	5.0 ± 6.3	93	15.7 ± 5.7	77	16.5 ± 8.4	78	19.5 ± 4.1	76	14.9 ± 7.3	197	12.3 ± 3.3	105	21.6 ± 8.7	216	14.7 ± 6.7
Cyenopyrafen	97	17.7 ± 6.2	129	7.0 ± 1.3	76	11.7 ± 1.3	111	12.6 ± 2.1	102	11.0 ± 3.7	372	1.4 ± 2.4	111	9.2 ± 2.5	80	3.7 ± 2.8
Cyflumetofen	177	11.9 ± 3.0	104	3.8 ± 0.7	86	9.3 ± 0.9	83	8.5 ± 2.5	113	7.6 ± 4.7	481	2.8 ± 2.1	96	3.3 ± 2.6	79	8.6 ± 4.4
Etoxazole	330	33.9 ± 4.0	126	0.0 ± 0.0	164	16.9 ± 6.1	320	0.0 ± 0.0	114	0.0 ± 0.0	262	0.0 ± 0.0	149	2.1 ± 2.2	463	3.0 ± 2.8
Fluxametamide	339	60.2 ± 3.9	172	16.5 ± 7.7	106	23.5 ± 4.9	128	48.2 ± 6.1	103	47.6 ± 5.2	405	41.4 ± 1.1	149	2.1 ± 2.2	80	24.0 ± 5.8
Pyflubumide	149	22.6 ± 4.9	109	6.5 ± 1.0	357	11.2 ± 1.6	94	12.3 ± 2.5	90	9.4 ± 3.2	457	3.6 ± 2.3	110	4.7 ± 1.9	113	6.4 ± 4.13
Pyridaben	308	63.5 ± 3.4	269	2.7 ± 2.2	431	0.0 ± 0.0	268	0.4 ± 0.8	285	2.1 ± 0.9	220	1.3 ± 1.3	323	2.1 ± 0.7	401	29.7 ± 10.2
Spirodiclofen	232	100.0 ± 0.0	94	82.7 ± 3.6	278	84.5 ± 5.7	101	91.1 ± 1.6	76	89.1 ± 5.1	462	85.0 ± 3.6	93	77.5 ± 4.3	102	100.0 ± 0.0
Spiromesifen	254	98.8 ± 0.1	116	82.8 ± 1.4	82	82.5 ± 1.7	94	95.8 ± 0.3	109	95.5 ± 2.1	467	94.8 ± 2.2	116	89.8 ± 5.2	94	89.8 ± 5.0
Spirotetramat	113	18.5 ± 6.2	153	7.9 ± 0.2	72	10.4 ± 1.0	114	13.9 ± 5.0	89	25.7 ± 3.7	314	0.0 ± 0.0	104	18.6 ± 2.5	127	0.0 ± 0.0

^a^ % Mortality stands for the % mortality at the field recommended dose.

**Table 7:** Mortality of *T. urticae* adults of eight field populations in response to 12 acaricides.

Acaricide	% Mortality ^a^ (mean ± SE)															
	n	CJ	n	GH-1	n	GH-2	n	GM-1	n	GM-2	n	OC	n	PT	n	YI
Abamectin	75	90.8 ± 2.0	81	16.5 ± 4.5	80	10.0 ± 5.8	84	13.7 ± 3.0	67	12.5 ± 3.0	80	13.3 ± 3.3	83	11.8 ± 2.6	80	24.7 ± 4.4
Acequinocyl	86	100.0 ± 0.0	65	6.4 ± 3.6	84	14.6 ± 7.9	98	2.6 ± 2.6	80	20.0 ± 5.1	96	10.7 ± 3.0	67	27.7 ± 1.4	92	50.0 ± 4.8
Bifenazate	76	73.3 ± 8.2	81	22.7 ± 3.7	78	19.9 ± 3.9	92	11.1 ± 2.8	81	23.0 ± 2.0	90	65.9 ± 6.5	80	6.7 ± 3.3	86	51.5 ± 3.0
Cyenopyrafen	76	78.5 ± 5.5	80	13.3 ± 3.3	86	9.1 ± 5.3	92	13.9 ± 2.8	80	11.7 ± 3.3	78	14.1 ± 7.1	88	13.9 ± 7.4	90	0.0 ± 0.0
Cyflumetofen	85	30.5 ± 4.4	86	38.7 ± 4.7	92	0.0 ± 0.0	86	11.9 ± 2.4	73	20.3 ± 5.9	82	18.7 ± 4.1	84	6.1 ± 6.1	80	10.0 ± 5.8
Etoxazole	75	0.2 ± 2.2	76	0.0 ± 0.0	75	0.0 ± 0.0	75	0.0 ± 0.0	70	0.0 ± 0.0	85	1.3 ± 3.9	64	0.0 ± 0.0	62	0.0 ± 0.0
Fluxametamide	81	94.3 ± 1.5	82	41.7 ± 4.8	70	52.4 ± 4.0	72	47.6 ± 2.4	71	43.1 ± 2.1	68	94.4 ± 5.6	98	25.6 ± 7.8	79	54.7 ± 1.6
Pyflubumide	82	13.0 ± 4.4	80	16.7 ± 4.8	86	0.0 ± 0.0	88	17.8 ± 4.9	74	5.4 ± 3.4	80	0.0 ± 0.0	82	6.7 ± 6.7	78	3.3 ± 3.3
Pyridaben	70	2.7 ± 2.7	75	2.6 ± 2.3	60	1.0 ± 3.3	63	2.3 ± 2.9	72	6.4 ± 3.2	68	3.5 ± 3.1	67	16.0 ± 4.3	67	5.3 ± 1.9
Spirodiclofen	86	2.1 ± 3.5	0	0.0 ± 0.0	88	5.4 ± 8.9	76	0.0 ± 0.0	80	8.3 ± 9.2	80	10.2 ± 7.1	83	6.3 ± 3.3	81	9.5 ± 6.9
Spiromesifen	83	3.5 ± 5.3	81	3.1 ± 1.6	80	0.0 ± 0.0	80	0.0 ± 0.0	90	5.4 ± 8.7	93	8.6 ± 5.1	81	3.1 ± 3.6	81	7.3 ± 8.4
Spirotetramat	76	29.7 ± 5.2	84	18.5 ± 5.0	84	0.0 ± 0.0	96	36.8 ± 4.8	75	12.6 ± 1.2	82	15.8 ± 8.2	80	20.0 ± 5.8	82	9.7 ± 0.3

^a^ % Mortality stands for the % mortality at the field recommended dose.

**Table 8:** Toxicity to etoxazole in susceptible and field-collected populations of *T. urticae* eggs.

Populations	n	%Mortality ^a^	LC_50_ (ppm a.i.) (95% CL ^b^)	Slope ± SE	RR ^c^
S	1780	100.0	0.02 (0.02–0.03)	1.95 ± 0.16	1.0
CJ	1647	33.9	>500	-	>25,500
GH-1	479	0.0	>500	-	>25,500
GH-2	904	19.6	>500	-	>25,500
GM-1	1687	0.0	>500	-	>25,500
GM-2	369	0.0	>500	-	>25,500
OC	1153	0.0	>500	-	>25,500
PT	339	2.1	>500	-	>25,500
YI	1127	3.0	>500	-	>25,500

^a^ % Mortality stands for the % mortality at field recommended dose. ^b^ Confidence limits. ^c^ RR represents resistance ratio = LC_50_ of resistant strain/LC_50_ of susceptible strain.

**Table 9:** Toxicity to pyridaben in susceptible and field-collected populations of *T. urticae* eggs.

Populations	n	%Mortality ^a^	LC_50_ (ppm a.i.) (95% CL ^b^)	Slope ± SE	RR ^c^
S	462	100.0	0.73 (0.31–1.48)	0.91 ± 0.10	1.0
CJ	2243	63.5	103.26 (76.59–150.66)	1.32 ± 0.12	144.5
GH-1	1449	2.7	>4000	-	>5480
GH-2	1819	0.0	>4000	-	>5480
GM-1	887	0.4	>4000	-	>5480
GM-2	778	2.1	>4000	-	>5480
OC	2227	1.3	>4000	-	>5480
PT	1073	2.1	>4000	-	>5480
YI	1533	29.7	>4000	-	>5480

^a^ % Mortality stands for the % mortality at field recommended dose. ^b^ Confidence limits. ^c^ RR represents resistance ratio = LC_50_ of resistant strain/LC_50_ of susceptible strain.

**Table 10:** Genotypes of point mutations in the CHS1 and PSST genes of *T. urticae*.

Population	n	Predominant Genotype	CHS1 Genotypes (%)		Predominant Genotype	PSST Genotypes (%)	
			I1017F			H92R	
			I	F		H	R
S	200	I	100.0	0.0	H	89.0	11.0
ER	200	F	0.0	100.0	H	71.0	29.0
PR	200	I	98.0	2.0	R	3.0	97.0
CJ	200	F	28.0	72.0	H	70.0	30.0
GH-1	200	F	9.0	91.0	R	13.0	87.0
GH-2	200	F	3.0	97.0	R	6.0	94.0
GM-1	200	F	1.0	99.0	R	8.0	92.0
GM-2	200	F	11.0	89.0	R	7.0	93.0
OC	200	F	20.0	80.0	H/R	63.0	37.0
PT	200	F	3.0	97.0	R	9.0	91.0
YI	200	F	15.0	85.0	H/R	55.0	45.0

## References

[1] Bolland H.R., Gutierrez J., Flechtmann C.H.W. (1998). World Catalogue of the Spider Mite Family (Acari: Tetranychidae).

[2] Choi K.M., Ahn S.B., Lee S.W., Lee M.H. (1989). Compendium of Insect Pests of Fruit Trees with Color Plates.

[3] Ilias A., Vontas J., Tsagkarakou A. (2014). Global distribution and origin of target site insecticide resistance mutations in *Tetranychus urticae*. Insect Biochem. Mol. Biol..

[4] Ilias A., Vassiliou V.A., Vontas J., Tsagkarakou A. (2017). Molecular diagnostics for detecting pyrethroid and abamectin resistance mutations in *Tetranychus urticae*. Pestic. Biochem. Physiol..

[5] Van Leeuwen T., Vontas J., Tsagkarakou A., Tirry L., Ishaaya I., Horowitz A.R. (2009). Mechanisms of acaricide resistance in the two-spotted spider mite *Tetranychus urticae*. Biorational Control of Arthropod Pests: Application and Resistance Management.

[6] Van Leeuwen T., Vontas J., Tsagkarakou A., Dermauw W., Tirry L. (2010). Acaricide resistance mechanisms in the two-spotted spider mite *Tetranychus urticae* and other important Acari: A review. Insect Biochem. Mol. Biol..

[7] Herron G.A., Woolley L.K., Langfield K.L., Chen Y. (2018). First detection of etoxazole resistance in Australian two-spotted mite *Tetranychus urticae* Koch (Acarina: Tetranychidae) via bioassay and DNA methods. Austral Entomol..

[8] Lee Y.S., Song M.H., Ahn K.S., Lee K.Y., Kim J.W., Kim G.H. (2003). Monitoring of acaricide resistance in two-spotted spider mite (*Tetranychus urticae*) populations from rose greenhouses in Korea. J. Asia-Pac. Entomol..

[9] Monteiro V.B., Gondim M.G.C., Oliveira J.E.M., Siqueira H.A.A., Sousa J.M. (2015). Monitoring *Tetranychus urticae* Koch (Acari: Tetranychidae) resistance to abamectin in vineyards in the Lower Middle São Francisco Valley. Crop Prot..

[10] Xu D., He Y., Zhang Y., Xie W., Wu Q., Wang S. (2018). Status of pesticide resistance and associated mutations in the two-spotted spider mite, *Tetranychus urticae*, in China. Pestic. Biochem. Physiol..

[11] Dekeyser M.A. (2005). Acaricide mode of action. Pest Manag. Sci..

[12] Suzuki J., Ishida T., Toda K., Ikeda T., Tsukidate Y., Kikuchi Y., Ito Y. (1989). Preparation of 2,4-diphenyloxazolines and—thia-zolines as ovicidal insecticides and acaricides. Eur. Pat. EP.

[13] Suzuki J., Ishida T., Kikuchi Y., Ito Y., Morikawa C., Tsukidate Y., Tanji I., Ota Y., Toda K. (2002). Synthesis and activity of novel acaricidal/insecticidal 2, 4-diphenyl-1, 3-oxazolines. J. Pestic. Sci..

[14] Van Leeuwen T., Demaeght P., Osborne E.J., Dermauw W., Gohlke S., Nauen R., Grbic M., Tirry L., Merzendorfer H., Clark R.M. (2012). Population bulk segregant mapping uncovers resistance mutations and the mode of action of a chitin synthesis inhibitor in arthropods. Proc. Natl. Acad. Sci. USA.

[15] Kobayashi M., Kobayashi S., Nishimori T. (2001). Occurrence of etoxazole resistance individuals of the two-spotted spider mite, *Tetranychus urticae* Koch from a limited region. Jpn. J. Appl. Entomol. Zool..

[16] Demaeght P., Osborne E.J., Odman-Naresh J., Grbić M., Nauen R., Merzendorfer H., Clark R.M., Van Leeuwen T. (2014). High resolution genetic mapping uncovers chitin synthase-1 as the target-site of the structurally diverse mite growth inhibitors clofentezine, hexythiazox and etoxazole in *Tetranychus urticae*. Insect Biochem. Mol. Biol..

[17] Osakabe M., Imamura T., Nakano R., Kamikawa S., Tadatsu M., Kunimoto Y., Doi M. (2017). Combination of restriction endonuclease digestion with the ΔΔCt method in real-time PCR to monitor etoxazole resistance allele frequency in the two-spotted spider mite. Pestic. Biochem. Physiol..

[18] Tirello P., Pozzebon A., Cassanelli S., Van Leeuwen T., Duso C. (2012). Resistance to acaricides in Italian strains of *Tetranychus urticae*: Toxicological and enzymatic assays. Exp. Appl. Acarol..

[19] Hollingworth R.M., Ahammadsahib K.I., Gadelhak G., McLaughlin J.L. (1994). New inhibitors of Complex I of the mitochondrial electron transport chain with activity as pesticides. Biochem. Soc. Trans..

[20] Stumpf N., Nauen R. (2001). Cross-resistance, inheritance, and biochemistry of mitochondrial electron transport inhibitor-acaricide resistance in *Tetranychus urticae* (Acari: Tetranychidae). J. Econ. Entomol..

[21] Hirata K., Kawamura Y., Kudo M., Igarashi H. (1995). Development of a new acaricide, pyridaben. J. Pestic. Sci..

[22] Devine G.J., Barber M., Denholm I. (2001). Incidence and inheritance of resistance to METI-acaricides in European strains of the two-spotted spider mite (*Tetranychus urticae*) (Acari: Tetranychidae). Pest Manag. Sci..

[23] Nauen R., Stumpf N., Elbert A., Zebitz C.P.W., Kraus W. (2001). Acaricide toxicity and resistance in larvae of different strains of *Tetranychus urticae* and *Panonychus ulmi* (Acari: Tetranychidae). Pest Manag. Sci..

[24] Khajehali J., Van Nieuwenhuyse P., Demaeght P., Tirry L., Van Leeuwen T. (2011). Acaricide resistance and resistance mechanisms in *Tetranychus urticae* populations from rose greenhouses in the Netherlands. Pest Manag. Sci..

[25] Sparks T.C., Nauen R. (2015). IRAC: Mode of action classification and insecticide resistance management. Pestic. Biochem. Physiol..

[26] Van Leeuwen T., Dermauw W., Grbic M., Tirry L., Feyereisen R. (2013). Spider mite control and resistance management: Does a genome help?. Pest Manag. Sci..

[27] Bajda S., Dermauw W., Panteleri R., Sugimoto N., Douris V., Tirry L., Osakabe M., Vontas J., Van Leeuwen T. (2017). A mutation in the PSST homologue of complex I (NADH: Ubiquinone oxidoreductase) from *Tetranychus urticae* is associated with resistance to METI acaricides. Insect Biochem. Mol. Biol..

[28] SAS Institute (2010). SAS/STAT User’s Guide: Statistics, Version 9.1.

[29] Choi J., Koo H.N., Kim S.I., Park B., Kim H., Kim G.H. (2020). Target-site mutations and glutathione S-transferases are associated with acequinocyl and pyridaben resistance in the two-spotted spider mite *Tetranychus urticae* (Acari: Tetranychidae). Insects.

[30] Lee S.Y., Ahn K.S., Kim C.S., Shin S.C., Kim G.H. (2004). Inheritance and stability of etoxazole resistance in twospotted spider mite. Tetranychus urticae, and its cross resistance. Korean J. Appl. Entomol..

[31] Fotoukkiaii S.M., Mermans C., Wybouw N., Van Leeuwen T. (2020). Resistance risk assessment of the novel Complex II inhibitor pyflubumide in the polyphagous pest *Tetranychus urticae*. J. Pest Sci..

[32] Ochiai N., Mizuno M., Mimori N., Miyake T., Dekeyser M., Canlas L.J., Takeda M. (2007). Toxicity of bifenazate and its principal active metabolite, diazene, to *Tetranychus urticae* and *Panonychus citri* and their relative toxicity to the predaceous mites, *Phytoseiulus persimilis* and *Neoseiulus californicus*. Exp. Appl. Acarol..

[33] Feng K., Ou S., Zhang P., Wen X., Shi L., Yang Y., Hu Y., Zhang Y., Shen G., Xu Z. (2019). The cytochrome P450 CYP389C16 contributes to the cross-resistance between cyflumetofen and pyridaben in *Tetranychus cinnabarinus* (Boisduval). Pest Manag. Sci..

[34] Salman S.Y., Aydınlı F., Ay R. (2015). Etoxazole resistance in predatory mite *Phytoseiulus persimilis* A.-H. (Acari: Phytoseiidae): Cross-resistance, inheritance and biochemical resistance mechanisms. Pestic. Biochem. Physiol..

[35] Ioriatti C., Anfora G., Angeli G., Mazzoni V., Trona F. (2009). Effects of chlorantraniliprole on eggs and larvae of *Lobesia botrana* (Denis & Schiffermüller) (Lepidoptera: Tortricidae). Pest Manag. Sci..

[36] Ouyang Y., Montez G.H., Liu L., Grafton-Cardwell E.E. (2012). Spirodiclofen and spirotetramat bioassays for monitoring resistance in citrus red mite, *Panonychus citri* (Acari: Tetranychidae). Pest Manag. Sci..

[37] Khalighi M., Tirry L., Van Leeuwen T. (2014). Cross-resistance risk of the novel complex II inhibitors cyenopyrafen and cyflumetofen in resistant strains of the two-spotted spider mite *Tetranychus urticae*. Pest Manag. Sci..

[38] Insecticide Resistance Action Committee (IRAC). http://www.irac-online.org/modes-of-action/.

[39] Fotoukkiaii S.M., Wybouw N., Kurlovs A.H., Tsakireli D., Pergantis S.A., Clark R.M., Vontas J., Van Leeuven T. (2021). High-resolution genetic mapping reveals cis-regulatory and copy number variation in loci associated with cytochrome P450-mediated detoxification in a generalist arthropod pest. PLoS Genet..

[40] Nauen R. (2005). Spirodiclofen: Mode of action and resistance risk assessment in tetranychid pest mites. J. Pestic. Sci..

[41] Marcic D., Mutavdzic S., Medjo I., Prijovic M., Peric L. (2011). Spirotetramat toxicity to immatures and sublethal effects on fecundity of female adults of *Tetranychus urticae* Koch. Zoosymposia.

[42] Cho J.R., Kim Y.J., Ahn Y.J., Yoo J.K., Lee J.O. (1995). Monitoring of acaricide resistance in field-collected populations of *Tetranychus urticae* (Acari: Tetranychidae) in Korea. Korean J. Appl. Entomol..

[43] Sugimoto N., Osakabe M. (2014). Cross-resistance between cyenopyrafen and pyridaben in the twospotted spider mite *Tetranychus urticae* (Acari: Tetranychidae). Pest Manag. Sci..

